# COVID-19 Drive-Through Point of Screening and Testing (POST) System: A Safe, Efficient, and Adaptable Model for Nasopharyngeal Swab Collection

**DOI:** 10.1017/dmp.2020.313

**Published:** 2020-09-02

**Authors:** Lauran K. Evans, Austin Shinagawa, Sarah Sutton, Lisa Calvo

**Affiliations:** 1 University of California, Los Angeles, Department of Head & Neck Surgery, Los Angeles, California; 2 University of Nevada, Reno School of Medicine, Reno, Nevada

**Keywords:** coronavirus, COVID-19, drive-through, health district, screening

## Abstract

**Objective:**

The authors aim to demonstrate that the current drive-through testing model at a health district was improved in certain parameters compared with a previous testing protocol, and to provide the methodology of the current model for other coronavirus disease (COVID-19) testing sites to potentially emulate.

**Methods:**

Initially, a small drive-through site was constructed at a converted tuberculosis clinic, but due to an increase in testing needs, an expanded point of screening and testing (POST) system was developed in an event center parking lot to administer tests to a higher volume of patients.

**Results:**

An average of 51.1 patients was tested each day (2.0 tests per personnel in personal protective equipment [PPE] per hour) at the initial tuberculosis clinic drive-through site, which increased to 217.8 patients tested each day (5.9 tests per personnel in PPE per hour) with the new drive-through POST system (*P* < 0.001). Mean testing time was 3.4 minutes and the total time on-site averaged 14.4 minutes.

**Conclusions:**

This POST drive-through system serves as an efficient, safe, and adaptable model for high volume COVID-19 nasopharyngeal swabbing that the authors recommend other COVID-19 testing sites nationwide consider adopting for their own use.

The novel severe acute respiratory syndrome coronavirus 2 (SARS-CoV-2) is the pathogen responsible for a pandemic beginning in 2020, which was declared an emergency by nearly every state in the United States.^1,2^ One of the challenges that health care providers are facing during this pandemic is inadequate access to diagnostic tests for patients.^3^ This not only presents the problem of the inability to confirm whether potential patients are positive for coronavirus disease (COVID-19), which could facilitate its spread,^4^ but it also distorts public opinion on the severity of this situation.^5^ In addition, patients with a confirmed diagnosis are more likely to adhere to self-quarantine orders,^6^ further preventing the spread of disease.

Increased testing also allows for public health officials to have a better understanding of this pandemic’s impact on the public. As the number of tests increases, so does the accuracy of statistical measures.^7^ An inadequate perspective on the prevalence of COVID-19 in communities could lead to the premature discontinuation of social distancing orders or recommendations, which then could cause a secondary peak in incidence rates. This trend was seen in St. Louis during the 1918 influenza pandemic,^8^ which outlines the importance of both accurate public perception of the pandemic’s severity and construction of strong epidemiological models. For these reasons, adequate availability and administration of diagnostic testing for COVID-19 are paramount for reducing further pandemic-related morbidity and mortality.

Specific sites for COVID-19 testing in several communities are necessary due to the scale of this pandemic. Emergency departments (EDs) in the United States, which are often already functioning near capacity under normal conditions,^9^ could be easily overwhelmed should patients present solely for COVID-19 testing purposes. Additionally, this could preclude social distancing, particularly in waiting rooms, and deplete personal protective equipment (PPE) and other resources in the hospital.^9,10^ Therefore, the installation of a specific testing program for COVID-19 could dramatically alleviate the burden of this pandemic on EDs.

The current gold standard diagnostic test is a reverse transcription polymerase chain reaction (RT-PCR), which detects viral RNA in respiratory secretions.^11^ One model described in the literature for the administration of this test, which typically uses nasopharyngeal and/or oropharyngeal swabs,^11^ is a drive-through site. This drive-through model, implemented at testing centers in several countries, allows for each patient’s automobile to function as an isolation compartment, preventing person-to-person spread at the site.^6-8,13^

In April 2020, the Washoe County Health District (WCHD) implemented a drive-through COVID-19 point of screening and testing (POST) system for the Reno-Sparks area community and surrounding rural areas, accounting for a total population of about 471 000. The POST system, which operates in the parking lot of an event center in Reno, Nevada, allows for the administration of hundreds of RT-PCR tests performed per testing day, at no cost to patients. This was developed to address limitations in daily test capacity, as well as inefficiencies at a previous testing station, which was converted from a pre-existing tuberculosis (TB) clinic.^10^ The authors hypothesize that this model is more successful in several parameters, such as total testing capacity and the number of tests completed at the drive-through, per tester in PPE per hour, compared with the previous testing center at the TB clinic. The authors also believe that the WCHD POST system, similar to those previously reported in the literature, represents a particularly efficient, safe, and adaptable model for COVID-19 testing, and recommend that other COVID-19 testing sites nationwide consider adopting it for their own purposes.

## METHODS

### COVID-19 Testing

Samples were obtained initially via both nasopharyngeal and oropharyngeal swabs in Phase 1 and nasopharyngeal swabs alone in the latter part of Phase 1 and throughout Phase 2. The CDC 2019-nCoV Real-Time RT-PCR Diagnostic Panel was used for the detection of COVID-19 when samples were sent to the state laboratory.

### TB Clinic Testing (Phase 1)

Starting March 6, 2020, the WCHD implemented Phase 1 of their testing protocol for potential COVID-19 patients. Patients were initially asked a series of questions relating to exposure, work environment, and symptoms by an assessment administered over the telephone through the WCHD COVID-19 Community Triage Line. Based on these questions, appointments were scheduled for patients deemed to be at high risk for infection. Patients with an appointment at this stage were likely receiving their first test; however, the differentiation between initial and follow-up testing for each patient was not recorded in the study. Testing during Phase 1 occurred at WCHD’s TB clinic, which was converted to a COVID-19 testing center. Testing occurred 6 days per week for 8 hours each day, from 9:00 am to 5:00 pm, with a variable number of patient appointments scheduled at each 60-minute interval. Swabs were obtained by a rotating group of 2 to 4 personnel in full PPE, while 1 staff member in partial PPE completed paperwork required for testing and prepared the specimens for shipment. Full PPE at this site entailed N95 masks, disposable gowns, disposable and reusable face shields, and nitrile gloves. Partial PPE entailed an N95 mask and gloves. The TB clinic functioned as a testing site for 21 days.

### POST System Testing (Phase 2)

**FIGURE 1:**
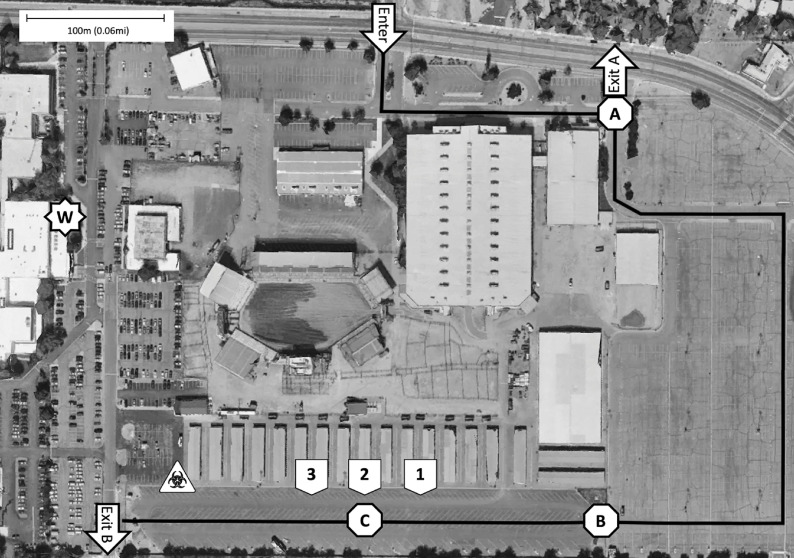
Schematic of the COVID-19 Testing Operation Conducted by the Health District and Located at an Adjacent Event Center. A, B, and C represent the 3 checkpoint stations where patients would stop their automobiles for check-in or testing; 1, 2, and 3 represent the testing stations, each staffed by 3 swabbing personnel. Exit A is an alternative exit for patients presenting without a confirmed appointment, and Exit B is the regular exit after testing. W represents the WCHD building, where the testers don, doff, and store PPE. The biohazard sign signifies the decontamination area for PPE. Map: Google, Maxar Technologies.^12^

On April 1, 2020, the WCHD implemented Phase 2 of their testing protocol, which consisted of the new POST system, located at the adjacent event center. Appointments were similarly determined by the phone triage line, so patients at highest risk were prioritized for testing. Testing in Phase 2 was conducted on Mondays, Wednesdays, Fridays, and Saturdays from 9:00 am to 1:00 pm with patients scheduled at each 15-minute interval. Workers in PPE generally arrived and exited the facility within 45–60 minutes of testing start and stop, for proper donning and doffing. Workers not in PPE usually arrived and exited the facility within 15–45 minutes of testing start and stop, for site setup and cleanup. The POST system is composed of multiple checkpoints that each automobile must progress through in sequential order. The specific layout of the WCHD POST system is detailed in Figure 1.

Patients determined to be high risk were scheduled via the WCHD COVID-19 Community Triage Line and provided the date and time of their drive-through appointment. The patients were instructed at this time to keep their windows fully closed when driving through the testing site, unless specifically directed otherwise by staff. The number of patients in each car ranged from 1–5 with most cars containing 1–2 patients. On the day of testing, automobiles that enter the site are directed to Checkpoint A. At this point, the automobile driver is instructed to display identification, through the window, for the person(s) receiving the test. Staff at Checkpoint A then confirm the appointment by matching the person(s) name and date of birth (DOB) to a list of scheduled tests for the day. If confirmed, an adhesive note with the patient’s “number,” assigned at the time of scheduling, is placed on the windshield (secured under the windshield wiper, if possible), and the patient is then directed to drive to Checkpoint B. If the appointment is not confirmed, the patient is provided a number to contact at the WCHD and is directed to leave the site through an alternative exit (see Figure 1).

There is a significant amount of distance delineated at the WCHD POST system from Checkpoint A to Checkpoint B (571 meters), allowing automobiles to line up while awaiting testing without disrupting the check-in process at Checkpoint A. Staff at Checkpoint B communicate the patient number on each windshield to an organizer located near the checkpoint. The organizer references this number to find the corresponding file containing a completed laboratory slip, consent form (Online Data Supplement 1), and stickers with the patient’s information. A clipboard containing these documents is handed to another staff member driving a golf cart, who will lead the patient to the appropriate testing station. Each golf cart, carrying the clipboards for 2 automobiles, leads these automobiles to 1 of 3 swabbing stations as soon as a station is available, before transferring the clipboard to the personnel involved in the swabbing. The automobile drivers are instructed to park their vehicles in the 2 designated parking spaces in front of each station.

Checkpoint C consists of the 3 swabbing stations covered by canopy tents, each staffed by 3 personnel wearing full PPE. Two of these personnel, labeled as “swabbers,” are each responsible for swabbing the patient(s) requiring testing in a single automobile. The third personnel in PPE acts as a “clerk,” whose responsibilities are recording the time of testing and which staff member swabbed which patient, preparing testing kits for the swabbers, attaching patient stickers to test tubes, and placing lab slips in the specimen bag. Once the 2 swab personnel each receive the appropriate clipboard from the golf cart driver, both hand the lab slip and stickers to the clerk before adding a new pen and COVID-19 informational sheet (Online Data Supplement 2) to the clipboard.

Then, swabbers approach their corresponding automobile with the clipboard and instruct the patients to open their window, which should have remained closed on the site prior to this point. The swabber verbally confirms the patient’s name and DOB and obtains the patient’s informed consent (Online Data Supplement 1) with signature following an explanation of the swabbing procedure. The patient is asked to keep the pen and informational sheet, while the clipboard containing the consent form is brought back to the station. Swabbers then obtain a testing kit from the clerk and again approach their respective vehicles. This testing kit contains a labeled test tube, nasopharyngeal swab, tissues, a paper cup, and hand sanitizer. Swabbers instruct patients to blow their nose and dispose of the tissue in the paper cup. Next, the swabber performs the nasopharyngeal swab in both nares before placing the sample in the test tube. The patient is given hand sanitizer, provided information regarding test follow-up along with direction on continued quarantine, and then directed to exit the testing site.

Once the 2 automobiles leave a station, the golf cart driver is signaled to lead the next pair of cars to that same station. During this transition, the swabber drops the sealed test tube into the appropriate specimen bag, held open by the clerk, who places the bag into an ice chest. Swabbers then change their gloves following every test to avoid cross-contamination. The ice chest containing patient specimens is transported to a nearby state health laboratory at 2-hour intervals during each shift by a health district employee. The WCHD call center or contact tracing staff notify the patients of their test results via phone or e-mail at approximately 48–72 hours after testing, answer any questions, and arrange for the next appropriate steps should the test be positive, including extensive contact tracing.

All personnel donning PPE at this location use a reusable heavy suit and hood powered air purifying respirator (PAPR), which are thoroughly sprayed before doffing with high-concentration ethanol solution (with or without bleach), following each shift, in the decontamination area (see Figure 1). Some other staff members also opted to use PPE while on-site, although this was not required.

### Data Collection and Analysis

Data collection ceased on April 27, 2020, for preparation of the current manuscript, although COVID-19 testing continues to be administered at this site. The following parameters were collected from the WCHD: COVID-19 tests performed each day, number of required staff and their responsibilities, positive COVID-19 tests per week, PPE use per shift, distances of the POST system route via measurement wheel, and safety concerns. The time intervals for individual automobiles driving through the site were recorded in a single day (April 27, 2020), although the researchers caution that the WCHD modified the testing hours from this day onward to decrease heat exposure for the workers. All other parameters this day were consistent with the rest of the POST system data set. The specific time intervals, measured in minutes, recorded for each automobile, included the exit from Checkpoint A, arrival at Checkpoint C, and exit from Checkpoint C. Descriptive statistics and 2-tailed independent sample t-tests were completed, comparing parameters at the TB clinic to those at the POST system. The number of swabs conducted per personnel in full PPE (as detailed previously) per hour was calculated as a measure of testing efficiency. Data were analyzed via SPSS, Version 26.0 (IBM Corp, Armonk, NY). Institutional Review Board determination/exemption was obtained through the University of Nevada, Reno.

## RESULTS

### Personnel and PPE

Swab personnel during Phase 1 who donned PPE consisted of WCHD registered nurses, per-diem nurses, third- or fourth-year medical students, and physicians. Phase 2 included the exact same swab personnel plus military medics, due to later involvement of the U.S. military. All swabbers in both phases were trained on donning, doffing, swabbing, and specimen handling protocols by a WCHD registered nurse. Support staff during Phase 2 consisted of sheriff’s office volunteers, fire department emergency medical technicians (EMTs), members of Team Rubicon (a veteran service organization), members of the Army and Air Force Reserves, Silver State Barricade and Sign employees, and WCHD employees. Phase 1 used the same support staff except for Team Rubicon, military, and the signage company, due to a lack of need for these services when the flow of cars was minimal. Support staff were trained in traffic safety and appropriate protocols regarding universal masking and COVID-19 safety.

Each day of testing at the TB clinic (Phase 1) required 4–8 personnel in full PPE (2–4 per half-day shift), averaging 6.14 personnel in full PPE per day. Other staff at the TB clinic included 1 clerk in partial PPE, 1–3 “flaggers” directing traffic, and 1 administrator handling paperwork. The PPE used at the TB clinic during each shift involved 1–2 N95 masks, 1–2 disposable gowns, and 1–2 disposable (or reusable) face shields per testing personnel. One to 2 pairs of nitrile gloves were used for each patient tested. The clerk in partial PPE used 1 N95 mask and approximately 10 pairs of gloves per shift.

At the POST system (Phase 2), there was an average of 9.23 personnel in full PPE per day. Other staff spread over the event center lot included 8–11 “flaggers” directing traffic, 5 golf cart drivers, 4–7 administrators handling paperwork, 1 safety officer, and 1–3 EMTs. The PPE at the POST system involved a total of 10 hood PAPRs, which were reused for the duration of the month; heavy suits for all testing personnel, replaced weekly; and 1–2 pairs of nitrile gloves per patient tested.

### Testing Metrics

**FIGURE 2:**
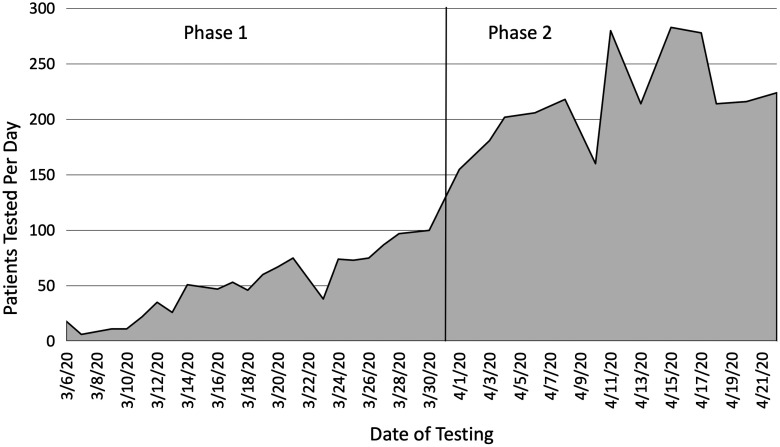
Number of Patients Tested Each Day at the Drive-through Testing Site Provided by the WCHD. The dates to the left of the line, prior to April 1, 2020, represent Phase 1 of testing conducted at the TB clinic. The dates to the right of the line, after April 1, 2020, represent Phase 2 of testing with the expanded POST system. Area under the curve represents all patients tested by the WCHD for COVID-19 until the cessation of data collection, involving 3903 patient tests in total.

A total of 1072 COVID-19 swab tests were conducted during Phase 1 at the TB clinic, over 21 days of active testing, with a mean of 51.1 (SD 28.8) patients tested per day. The positive COVID-19 cases tested at the TB clinic totaled 24 patients (2.2% of all patients tested). Phase 2 at the POST system yielded 2381 swab tests over 13 days of active testing, with a mean of 217.8 (SD 41.7) patients tested per day (Figure 2). The positive COVID-19 cases tested at the POST system, with positivity data limited to the first 9 days of testing at the time that data collection ceased, totaled 219 patients (11.5%). The maximum number of tests performed in 1 shift with the POST system was 283. Significantly more patients were tested per day at the POST system when compared with the TB clinic (*t*_32_ = −13.8, *P* < 0.001). At the TB clinic, there were 6.4 tests conducted per hour compared with 54.4 tests per hour at the POST system. In addition, while there were 2.0 tests, per personnel in full PPE, per hour administered at the TB clinic, there were 5.9 tests, per personnel in full PPE, per hour administered at the POST system (*t*_32_ = −11.74, *P* < 0.001).

### POST System Time Intervals

On April 27, 2020, patients in 136 automobiles were tested for COVID-19 during a 2-hour shift. The “total time on site,” defined as completion at Checkpoint A to completion at Checkpoint C, ranged from 8 to 20 minutes, with an average of 14.4 minutes. The “check-in time,” defined as Checkpoint A exit to Checkpoint C arrival, ranged from 4 to 17 minutes with an average of 11.0 minutes. The “testing time,” defined as Checkpoint C arrival to Checkpoint C exit, ranged from 2 to 6 minutes, 3.4 minutes on average. These time intervals are outlined in Table 1. The entire distance of the driving route through the WCHD POST system measured 999 meters (0.62 miles).

**TABLE 1:** Automobile Time Intervals Through Site Checkpoints

Time Interval	Start Time	End Time	Mean Time (mins)	Minimum Time (mins)	Maximum Time (mins)	SD	Associated Distance (meters)
Check-in time	A exit	C arrival	11.0	4	17	2.5	710
Testing time	C arrival	C exit	3.4	2	6	0.8	99
Total time on-site	A exit	C exit	14.4	8	20	2.5	809

*Check-in time* represents the exit from Checkpoint A (near entrance) to arrival to Checkpoint C (testing area). *Testing time* represents the arrival at Checkpoint C to the exit from Checkpoint C. *Total time on-site* represents the summation of the check-in time and testing time. All data were recorded on a single selected day of COVID-19 testing at the health district POST during Phase 2. Time was measured in whole minutes (mins) and distance in meters. SD = standard deviation.

### Safety Outcomes

There were 2 instances reported of a mismatched lab slip and test tube vial arriving at the laboratory during Phase 2. No instances were reported of a break in PPE or accidental contagion exposures among the staff. The largest safety concern at the POST system was the amount of time spent wearing the PAPR and heavy suit, considering the progressively increasing outdoor temperatures in Reno, particularly near the end of each shift at 1:00 pm. EMTs and safety officers were on-site at all times to address the safety of the patients and staff. Automobile safety was encouraged by advising personnel to walk behind vehicles and through constant communication with automobile drivers by “flaggers” and golf cart drivers.

## DISCUSSION

This drive-through POST system at a health department in the United States, which operates as a unified and efficient testing site available to both urban and rural populations, is an alternative to similar COVID-19 drive-through testing models previously reported in the literature.^6-8,13^ Key distinct features of the POST system are a clear and efficient layout with the capacity to test thousands daily, the implementation of a strict protocol for all staff on-site, and the use of trained professionals for obtainment of nasopharyngeal swabs. The POST system referenced in the current study was already able to accommodate a large volume of patients in a short time span, with up to 283 samples collected in 4 hours. This is comparable to the model averaging 192 patients per day reported by Ton et al.,^6^ as well as the model in South Korea, which could accommodate around 100 tests per day.^13^ However, the authors believe that the POST system is amenable to even further expansion. Recruitment of additional staff, parallel driving lanes, increased swabbing stations, longer site hours, and more testing days are all measures that could realistically expand the capacity of the POST system to thousands of patient tests per day.

The efficiency of the POST system should be highlighted as well. Two of the main limitations in the administration of high-volume COVID-19 testing are the availability of qualified personnel for swabbing (eg, nurses, medical students, and physicians) and the nationwide PPE shortage.^14,15^ Therefore, the authors measured the efficiency of both testing systems (Phases 1 and 2) through calculating the number of tests conducted in 1 hour by 1 personnel donned in full PPE. Results revealed 2 tests/PPE/hour for the TB clinic in Phase 1 and nearly 6 tests/PPE/hour in Phase 2. The authors did not identify a similar metric in the current literature for COVID-19 testing models and suggested that “COVID-19 tests/PPE/hour” could be used as an objective measure of testing model efficiency. Nevertheless, the authors believe that a single worker in PPE conducting nearly 6 tests per hour (or about 1 test per 10 minutes) is a satisfactory level of efficiency for a drive-through testing station. Additionally, the shortage of PPE was addressed in the WCHD POST system by the use of a PAPR and heavy suit. The PAPR allowed for ~100% efficiency at filtering air, which is more protective than the N95 masks used in other models.^14^ The reusable nature of both the PAPR and suit following decontamination significantly reduced the burden of this testing site in terms of PPE use relative to other models.^6^

Another set of metrics suggestive of the high efficiency of the POST system is the time intervals recorded throughout 1 day of testing. The total time on-site averaged around 14 minutes, consisting of about 11 minutes for check-in and 3 minutes for swabbing. These numbers are comparable to the model in South Korea, which references a specimen collection time of 2 minutes and total time of < 15 minutes.^7^ These short-time intervals not only considerably contribute to the efficiency and capacity of the POST system, but also reduce the associated time burden on patients’ schedules.

The authors also posit that the POST system is highly adaptable to communities in the United States, including areas with a larger or smaller population than the Reno-Sparks area. This model could be implemented in any comparably sized lot, although event centers are especially applicable due to their probable disuse for regular activity during this pandemic. The identification of patients at high risk for infection over a telephone triage line allows for the remote assessment of patients by physicians or epidemiologists, increasing the applicability of the POST system to rural areas, provided that a laboratory is within acceptable distance. However, patients should be strongly cautioned that serious symptoms should preclude testing through the POST system and instead prompt an in-person visit with a physician.

One obvious limitation of the POST system is that it is only available to patients with access to an automobile. Patients might also forego social distancing to access an automobile for testing purposes, such as asking a friend to drive them through the testing site. To accommodate patients without a car, the WCHD launched a mobile testing program with local emergency medical services. Patients requiring in-person testing were able to make appointments, via phone, for testing to be brought to their place of dwelling through this initiative. However, risk assessment was only available through online or telephone, and in-person referral was completed through other community testing sites, and not the currently described system. Another limitation includes the lack of a physician present on-site in the case of a medical emergency among staff or patients (eg, heat stroke or respiratory failure); however, a safety officer, emergency medical technicians, military medics, and registered nurses were all on-site who could help address potential emergencies. Because the stations in the POST system were covered by canopy tents, the swabbers were exposed to outside weather conditions. The testing schedule at the WCHD site was transitioned to start 1 hour earlier and remain open for 3 hours each day (after cessation of patient testing data collection), with the addition of a fourth testing station, to address concerns of heat and dehydration among swabbers. Similar problems were reported in the drive-through model in South Korea.^13^ This limitation might be alleviated by more protective tents or similar structures.

Serum COVID-19 antibody testing via antecubital venipuncture or finger prick could also be integrated into the POST system if such tests become widely available and are clinically indicated.^3^ Antibody drive-through testing may not necessitate the same degree of PPE as the nasopharyngeal swab, considering the decreased droplet and airborne risk. Additional considerations are inclusive of phlebotomists or further training of personnel, patient positioning for obtainment of serum sample in an automobile, the risk of bloodborne pathogen exposure, and the requirement for additional supplies. Previous processes from the current POST system that could transition effectively to antibody sample collection include specimen labeling, storage, and transport, as well as the telephone assessment and subsequent patient scheduling.

Translating this protocol for drive-through COVID-19 testing to other sites nationwide could significantly improve testing efficiency and reduce consumption of PPE for testing purposes. The authors believe that the POST system is an effective model for high-volume, safe, and efficient testing that is adaptable to most communities in the United States, and that it should be emulated in areas with inadequate testing programs.

## CONCLUSION

The POST system described in the present study improves on a prior testing center used by the health district, and represents a particularly efficient, safe, and adaptable model for COVID-19 testing. Although the limitations of this model should be considered before implementation, the authors recommend that other COVID-19 testing sites nationwide consider adopting it for their own purposes.
